# Ironing out sex differences in tuberculosis prevalence

**DOI:** 10.5588/ijtld.17.0194

**Published:** 2017-05-01

**Authors:** Tom A. Yates, Sarah H. Atkinson

**Affiliations:** * Institute for Global Health, University College London, London, UK; † KEMRI-Wellcome Trust Research Programme, Kilifi, Kenya; ‡ Department of Paediatrics, Oxford University Hospitals, University of Oxford, Oxford, UK, e-mail: t.yates@ucl.ac.uk

LAST YEAR, TWO STRIKING STUDIES focused attention on sex differences in tuberculosis (TB) epidemiology. Katherine Horton and team's meta-analysis of 56 prevalence surveys undertaken in low-and middle-income countries found that adult men had 2.21 (95% CI, 1.92–2.54) times more bacteriologically confirmed TB than adult women.^1^ The equivalent ratio for smear-positive TB (reported in 40 surveys), was 2.51 (95%CI 2.07–3.04). A male preponderance of prevalent TB was observed in all World Health Organization geographical regions that contributed sufficient data to draw meaningful conclusions. In the second study, Pete Dodd and colleagues utilised data on adult TB prevalence, the incidence of Mycobacterium tuberculosis infection in children, and data on social contact patterns from communities in Zambia and the Western Cape of South Africa, to produce modelled estimates of the incidence of M. tuberculosis infection, by age and sex.^2^ Whilst men and women had similar numbers of social contacts, contact patterns were strongly age and sex assortative. A key finding was that 66% (95%CI 64–67, Zambia) and 57% (95%CI 56–58, Western Cape) of incident M. tuberculosis infections were attributable to contact with adult men. The authors commented that this ‘was largely because tuberculosis prevalence was higher in male interviewees’.

There are a number of reasons why men might suffer a higher burden of TB. Horton et al. found that the ratio of prevalent to notified cases was 1.55-fold higher in men (95%CI 1.25–1.91), suggesting that men present to care later than women.^1^ Proximal risk factors for TB disease, such as smoking and drinking, tend to be more common in men, although other risk factors, such as exposure to indoor air pollution and, in some communities, human immunodeficiency virus (HIV) infection, more often affect women. M. tuberculosis is unusual in being a truly airborne pathogen, rather than one spread via respiratory droplets, so men's larger lung volumes may place them at greater risk. Immunological reasons for the excess of TB disease in men have also been proposed.^3^ The impact on TB prevalence of any differences by sex in susceptibility or disease duration would be amplified by social contact patterns.^2^ We propose that a further explanation for the greater burden of TB experienced by men may be sex-specific differences in iron metabolism.

Iron is critical for the growth of many pathogens. Invading pathogens utilise various strategies to access their hosts' iron stores, and sequestration of iron in response to inflammation is an important host defence against infectious diseases.^4^,^5^ A key mediator of this response is the hormone hepcidin, which reduces plasma iron levels by blocking both iron absorption in the duodenum as well as the egress of iron into the plasma from hepatocytes and macrophages.^4^ Erythropoesis inhibits hepcidin production, whereas high levels of iron in the plasma and the acute inflammatory response stimulate hepcidin production.^4^ High concentrations of hepcidin are associated with iron loading within macrophages. Hepcidin might therefore promote the growth of pathogens that reside within macrophages, such as Salmonella species and M. tuberculosis.^4–6^

Host iron status and hepcidin levels may predict TB disease in healthy individuals. Post mortem data from the 1920s suggested that adults with ‘iron overload’ were at greatly increased risk of death from TB.^7^ It is unclear whether this mortality was a result of higher TB incidence or higher case fatality rates, and the association may be confounded by alcohol intake. In Gambian studies, high baseline hepcidin concentrations have been associated with incident TB disease in 196 adults with HIV^8^ and in 27 adult household TB contacts.^9^ Small numbers and potential reverse causation, however, mean that these data should be interpreted cautiously. In another Gambian study, baseline iron status was associated with incident TB disease in a cohort of 1139 newly diagnosed HIV-positive adults.^10^ However, again, given the long incubation period of M. tuberculosis, reverse causation might explain this result. TB disease is known to cause anaemia, and this is thought to be mediated by hepcidin.^11^ However, in support of a causal association between iron levels within macrophages and TB disease, polymorphisms in the SLC11A1 gene, a cation transporter protein, are associated with TB susceptibility in African and Asian populations.^12^

Interestingly, hepcidin concentrations vary substantially by age and sex. Data from the Netherlands^13^ and Italy^14^ show that, in men, levels of the hormone rise with age and are more than twice the levels observed in premenopausal women. This is probably largely because, given the absence of menstrual blood loss, plasma iron levels tend to be higher in men. In Horton et al.'s meta-analysis, it is notable that the male to female ratio in prevalent TB, which increases with age, seems to level off as women reach the age of menopause.^1^ This pattern is entirely consistent with the hepcidin hypothesis. Given that iron status is potentially modifiable, the interactions between iron status, sex and TB merit further investigation.
